# Supplementary material for: Snap29 mutant mice recapitulate neurological and ophthalmological abnormalities associated with 22q11 and CEDNIK syndrome
Source: Commun Biol. 2019 Oct 11;2:375. doi: 10.1038/s42003-019-0601-5 (PMC6789041; doi:10.1038/s42003-019-0601-5)
Supplement: Supplementary file 1 — Supplementary Information [file 42003_2019_601_MOESM1_ESM.pdf]

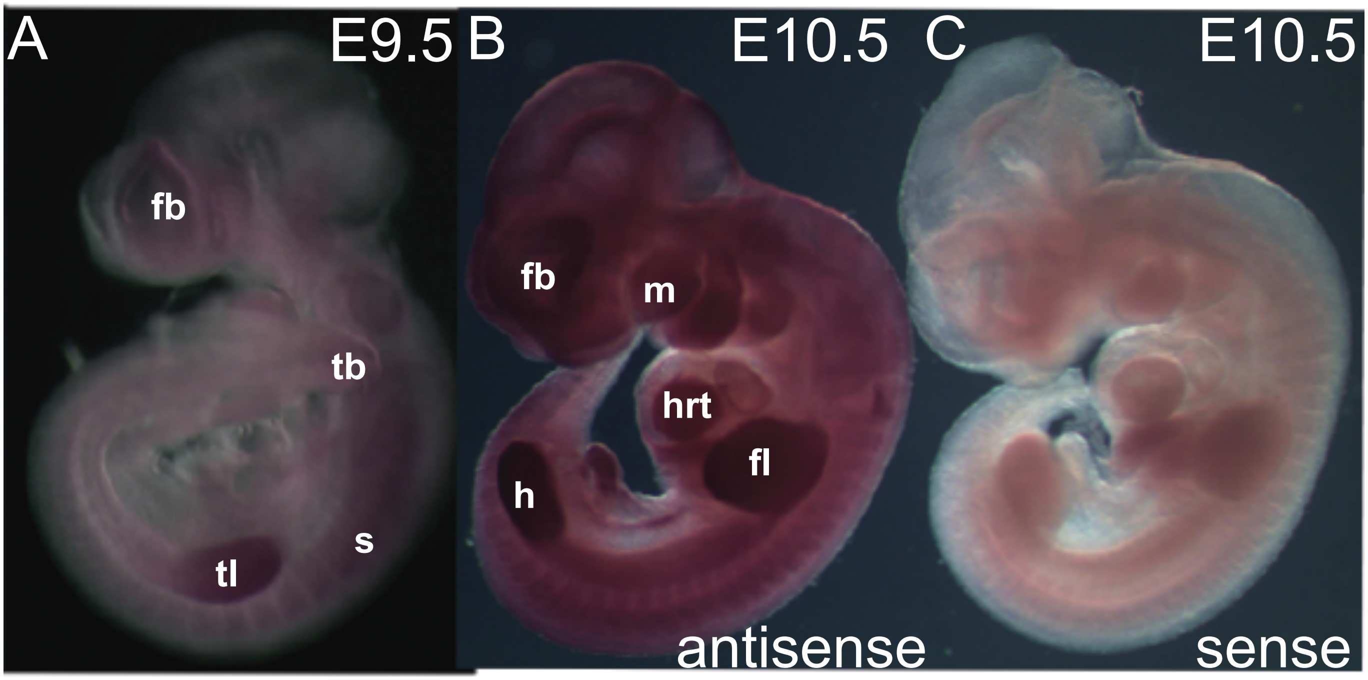

Supplementary Figure 1. ***Snap29*** **mRNA expression during embryogenesis.**
A. Expression of *Snap29* mRNA is expressed throughout the embryo at E9.5 including the somites and limb buds. **B.** By E10.5, expression is detectable everywhere but is most prominent in head tissues and limb buds. **C.** An embryo at E10.5 incubated with the sense probe is shown as a control.Wild-type is denoted by +/+, heterozygotes +/- and homozygous mutants by -/-. Lb: limbud, fore or hind, l: lung, hrt: heart, s: somite, tb: tailbud.

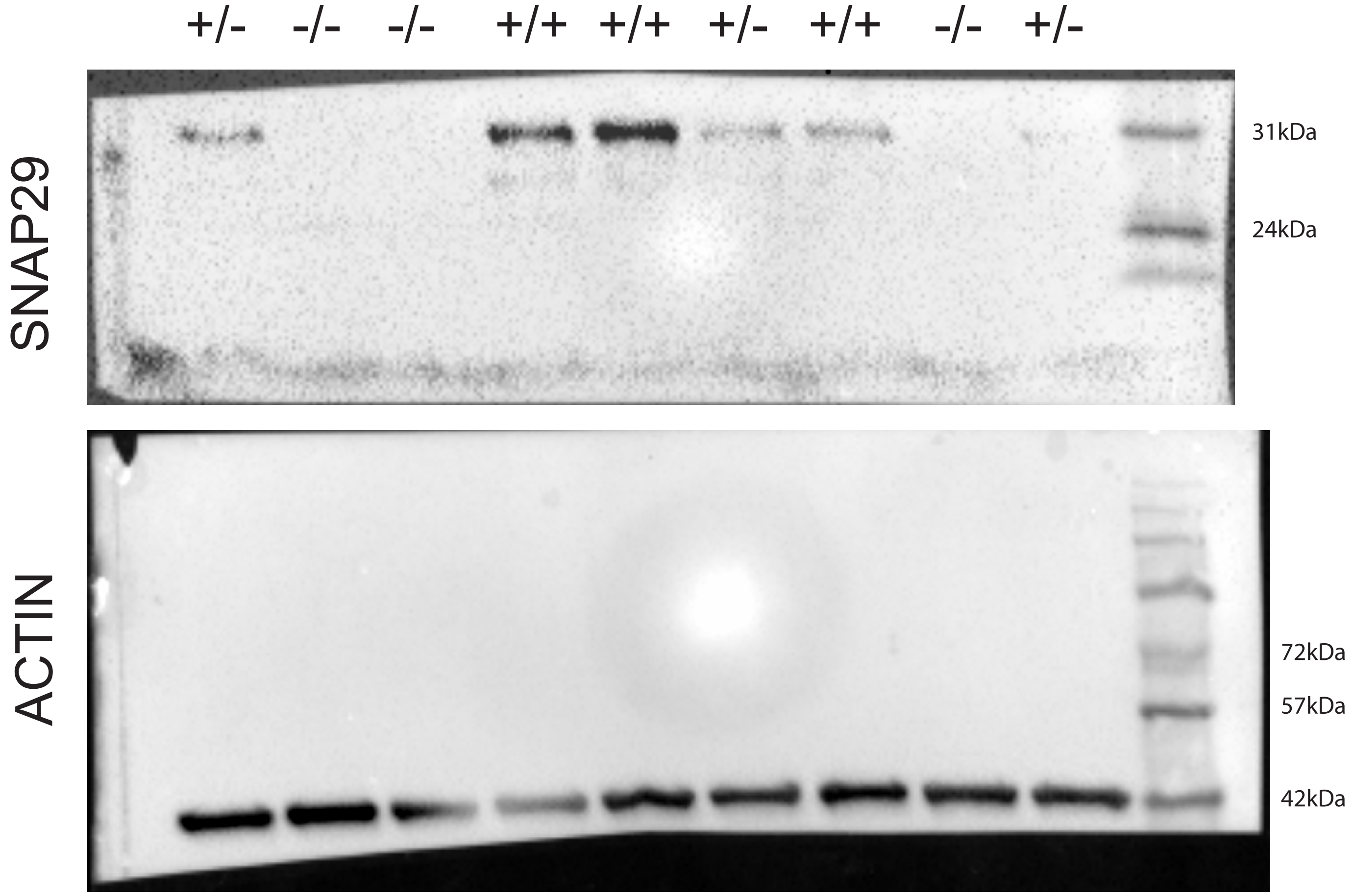

Supplementary Figure 2. SNAP29 levels in mice carrying the *Snap29* allele.
Complete western blot shown in figure 1C.

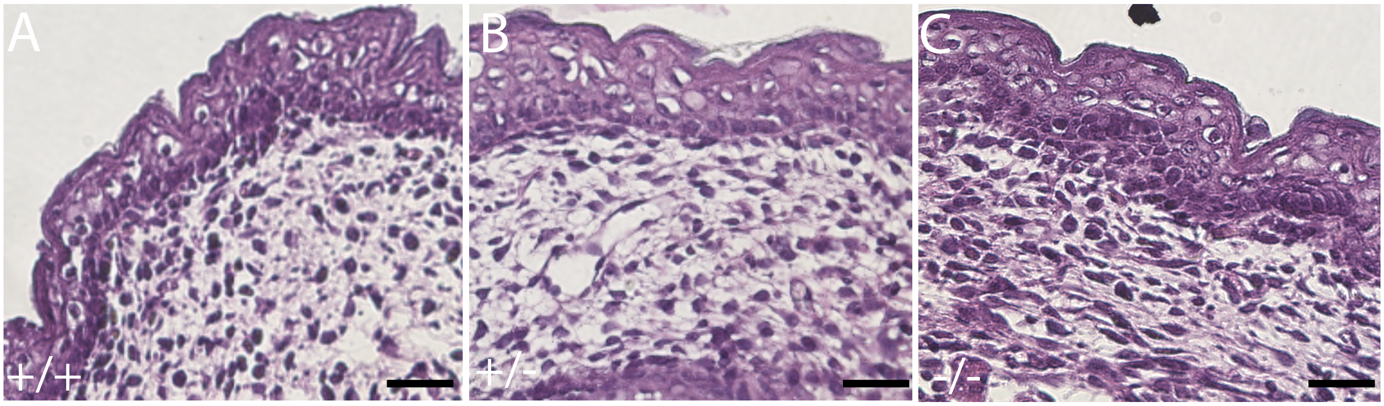

Supplementary Figure 3. *Snap29* mutant show no morphological defect at E16.5
Hematoxylin and eosin staining (A-C) of dorsal skin. No difference were observed between *Snap29*$^{+/+}$ (A), *Snap29*$^{+/-}$ (B) and *Snap29*$^{-/-}$ (C). Scale bars represent 25µm.

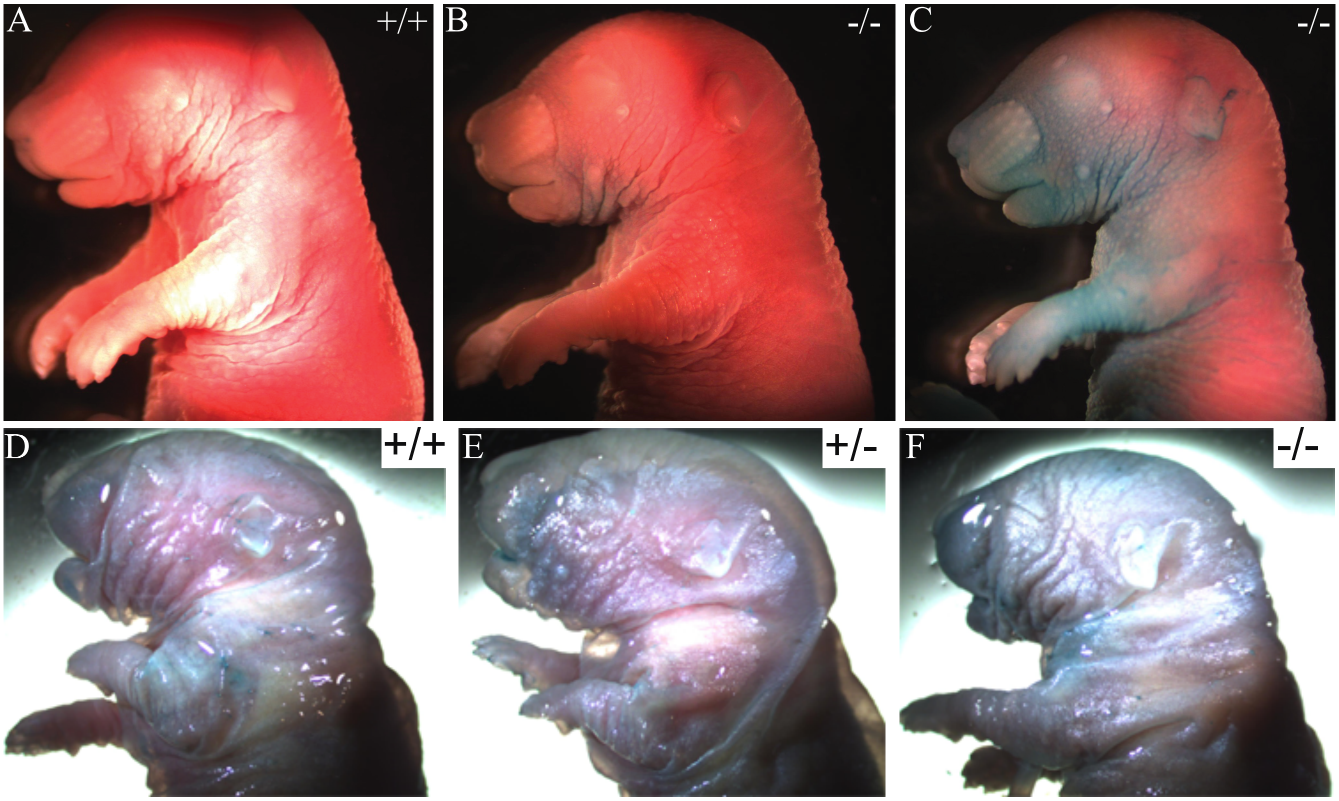

Supplementary Figure 4. *Snap29* mutant mice show delayed skin barrier formation.
X-Gal permeability assay at E17.5. Wild type embryos (A) and some *Snap29*$^{-/-}$ (B) embryos have no coloration suggesting that their skin was not permeable to X-Gal. However, some *Snap29*$^{-/-}$ E17.5 embryos (C) showed X-Gal staining on the ventral body wall, suggestive of a delay in skin barrier formation. By the P1 stage, no X-Gal staining is observed in *Snap29*$^{-/-}$(F) when compared to *Snap29*$^{+/+}$ (D) and *Snap29*$^{+/-}$ (E), suggesting that barrier formation is no longer delayed.

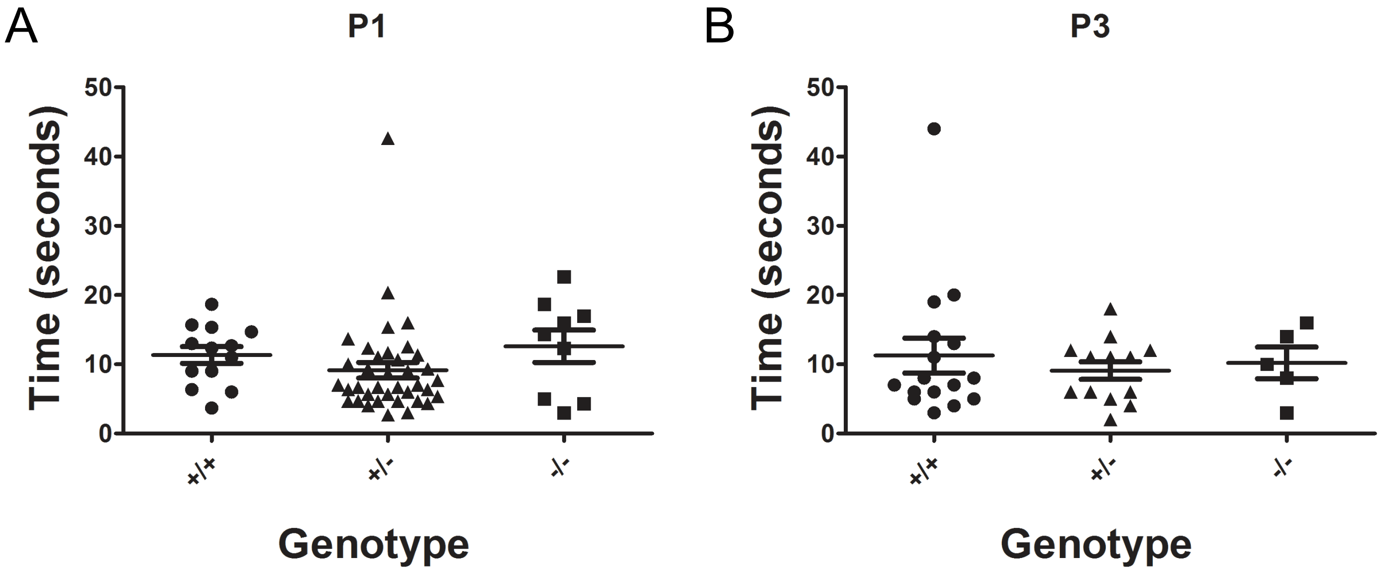

Supplementary Figure 5. *Snap29* mutant pups move slower than their sibling.
The time it takes to turn back on their paws when placed on their back in P1 pups (A) and P3 pups (B). *Snap29* homozygous mutant P1 pups turn slightly slower than their sibling although this difference is not significant. By P3, pups turned at the same rate independently of genotype. Error bars represent S.E.M.

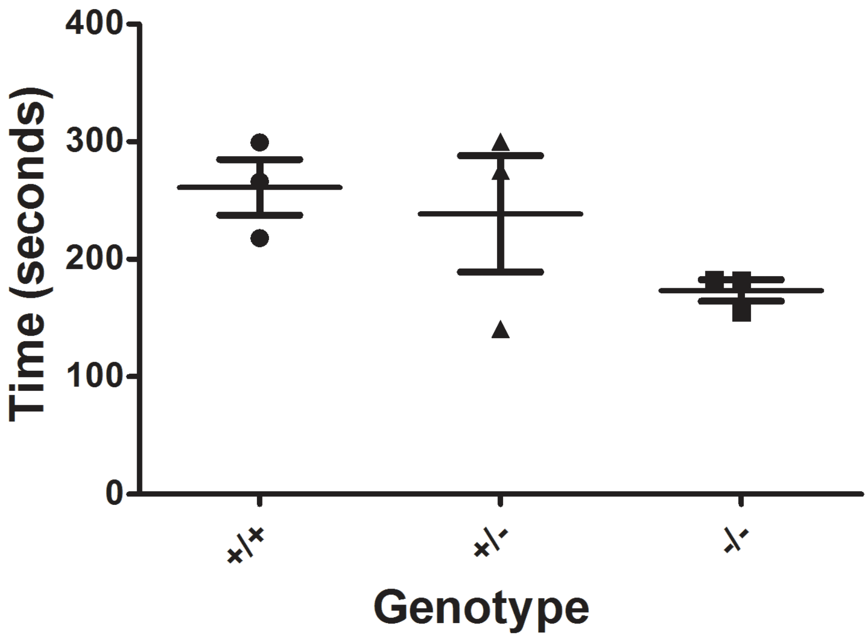

Supplementary Figure 6. *Snap29* mutant animal show a reduced latency to fall. Latency to fall as measured by rotarod assay is more pronounced in *Snap29*$^{-/-}$ male mice, although this result is not significant. Error bars represent S.E.M.

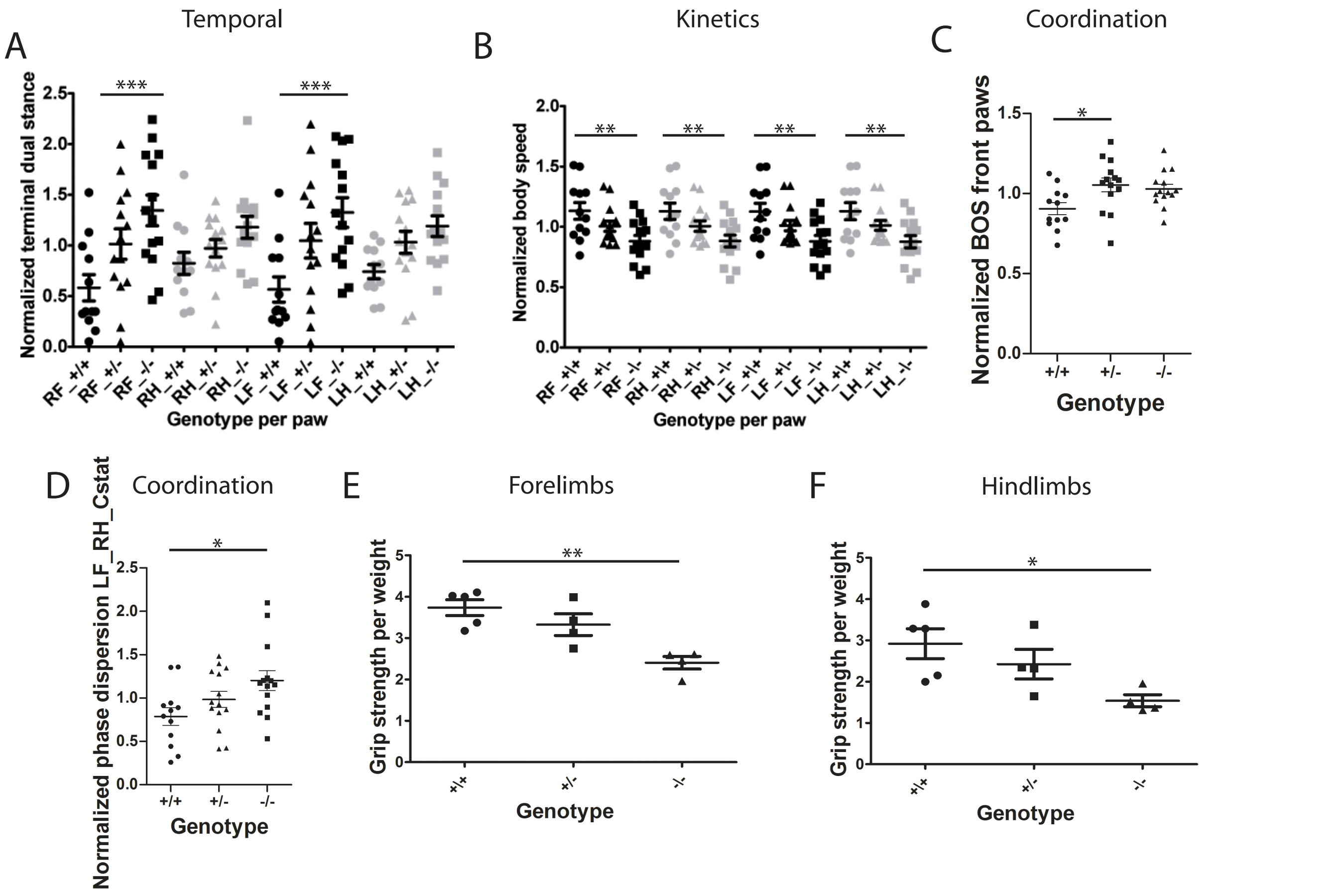

Supplementary Figure 7. Catwalk gait parameters that were affected in *Snap29*$^{-/-}$ females.
Catwalk assay was used to monitor gait parameters in *Snap29*$^{+/+}$, *Snap29*$^{+/-}$ and *Snap29*$^{-/-}$ females. The temporal parameter terminal dual stance was significantly elevated in both front paws (A). The kinetic parameter body speed was decreased in all paws of mutant animals (B). The interlimb coordination parameter BOS front paw was significantly increased in *Snap29*$^{+/-}$ females when compared to *Snap29*$^{+/+}$ animals (C). The interlimb coordination parameters phase dispersion LF_RH_Cstat was elevated in mutant females (D). The grip strength was assessed in 14 weeks old female for forelimbs (E) and hindlimbs (F). The reduce grip strength did not recover over time in *Snap29*$^{-/-}$ females. RF: right front paw; RH: right hind paw; LF: left front paw and LH: left hind paw. RF and LF are in black and RH and LH are in gray. Statistical significance: *: p<0.05, **: p<0.01 and ***: p<0.001. Error bars represent S.E.M.

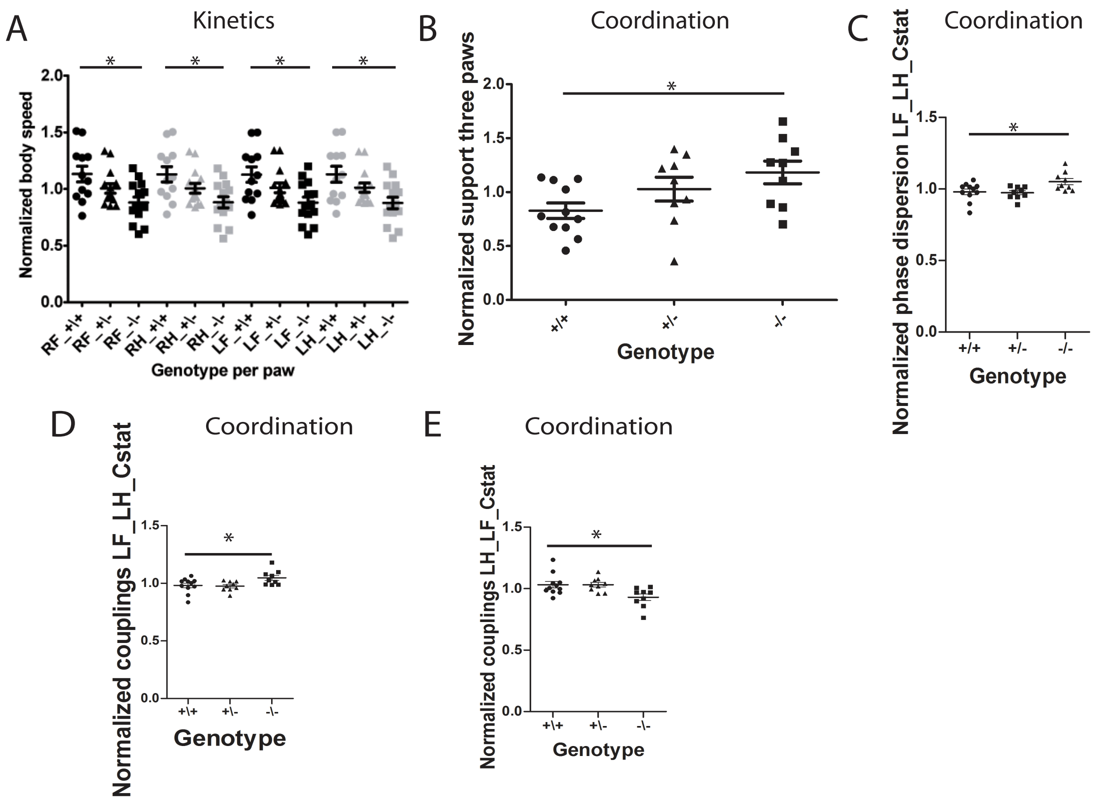

Supplementary Figure 8. Catwalk gait parameters that were changed in *Snap29*$^{-/-}$ males.
Catwalk assay was used to monitor gait parameters in *Snap29*$^{+/+}$, *Snap29*$^{+/-}$ and *Snap29*$^{-/-}$ males. The kinetic parameter body speed was decreased for all paws of *Snap29*$^{-/-}$ males (A). The interlimb coordination parameters support on three paws, phase dispersion LF_LH_Cstat and couplings LF_LH_Cstat were significantly elevated in *Snap29*$^{-/-}$ males (B,C and E). In contrast, the interlimb coordination parameter couplings LH_LF_Cstat was reduced in *Snap29*$^{-/-}$ males (D). RF: right front paw; RH: right hind paw; LF: left front paw and LH: left hind paw. RF and LF are in black and RH and LH are in gray. Statistical significance: *: p<0.05, **: p<0.01 and ***: p<0.001. Error bars represent S.E.M.

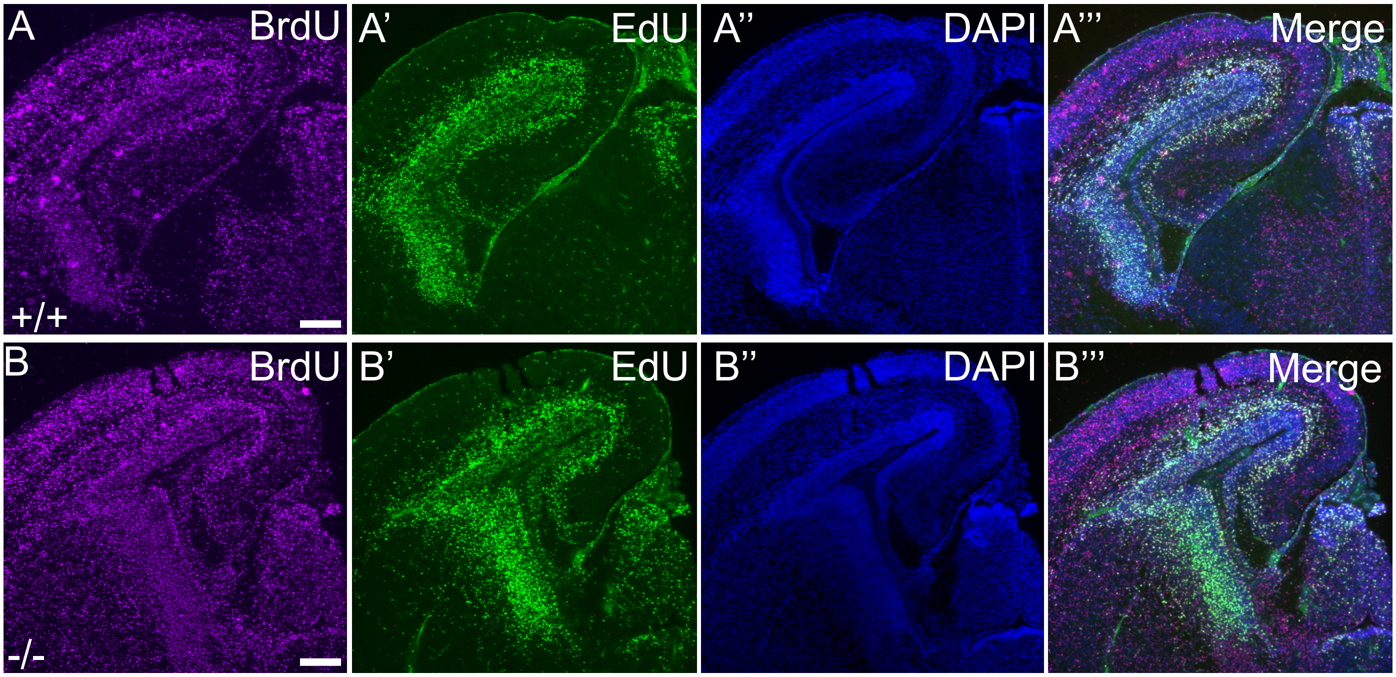

Supplementary Figure 9. Snap29 E17.5 mutant brains show increased proliferation.
E17.5 brain of *Snap29*$^{+/+}$ (A-A''') and *Snap29*$^{-/-}$ (B-B''') mice. BrdU is shown in red (A, B), EdU is green (A'-B'), the nuclear marker DAPI in blue (A'', B'') and the merge images is shown last (A'''-B'''). BrdU was injected first at E13.5 and EdU was injected at E15.5 prior to dissection. We observed and increase in BrdU and EdU signals in the brain of *Snap29*$^{-/-}$ brain suggesting increased proliferation is occurring. Scale bars represent 200μm.

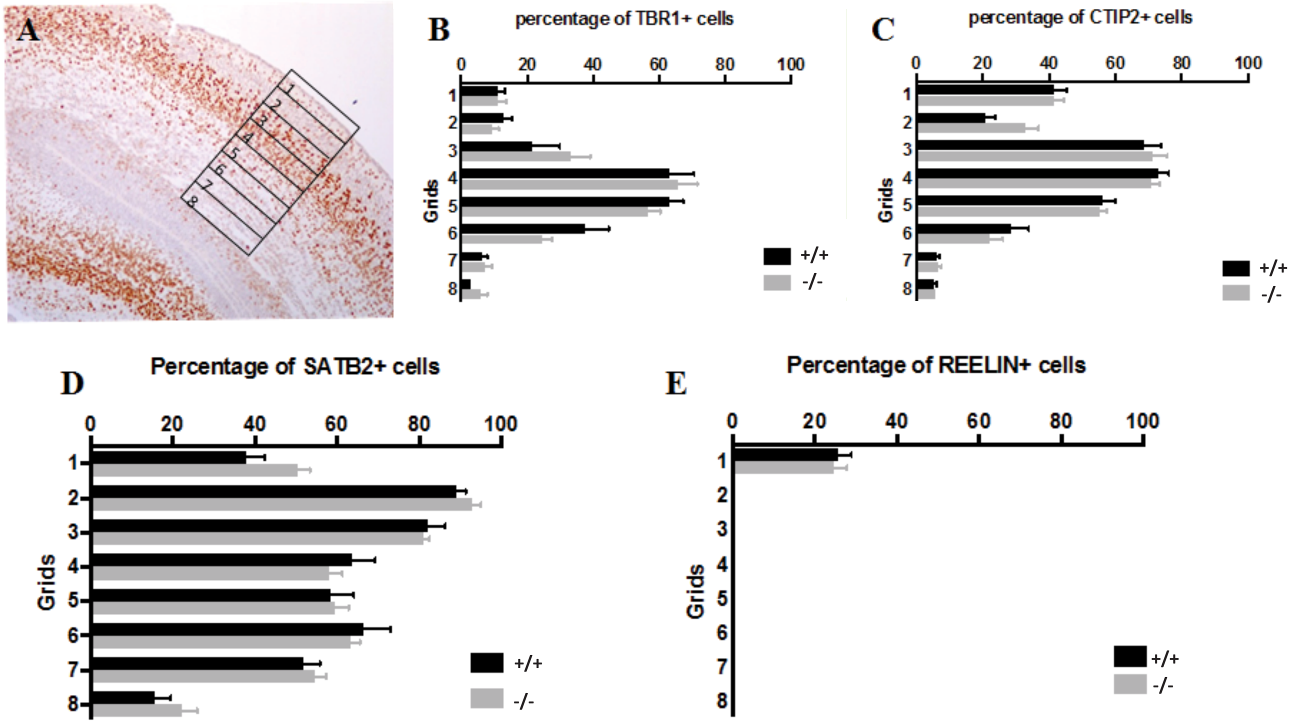

Supplementary Figure 10. Counting of cortical layer organization markers.
A. Representative image showing cell counting. B. Counting of Tbr1; C. Stip2; D. Satb2; E. Reelin stained neurons did not show differences between the *Snap29* genotypes. The total number of cells was counted using automatic local threshold (otsu, R:10). Error bars represent S.D.

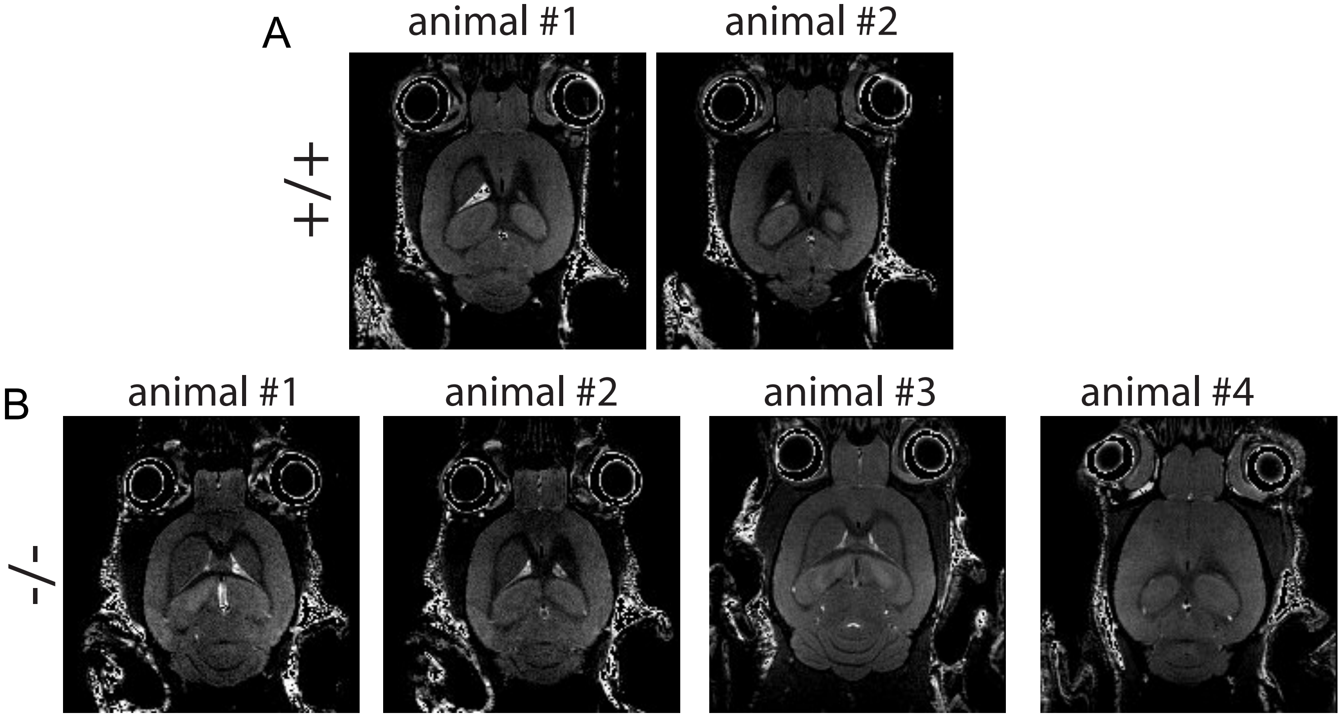

Supplementary Figure 11. MRI analysis of brain of *Snap29*$^{+/+}$ and *Snap29*$^{-/-}$ animals.
Representative layers of the head of 11 weeks old animal. Two wild type animals (A) and 4 homozygous mutant animals (B) were analyzed. The MRI revealed no differences between wild type and homozygous mutant animals.

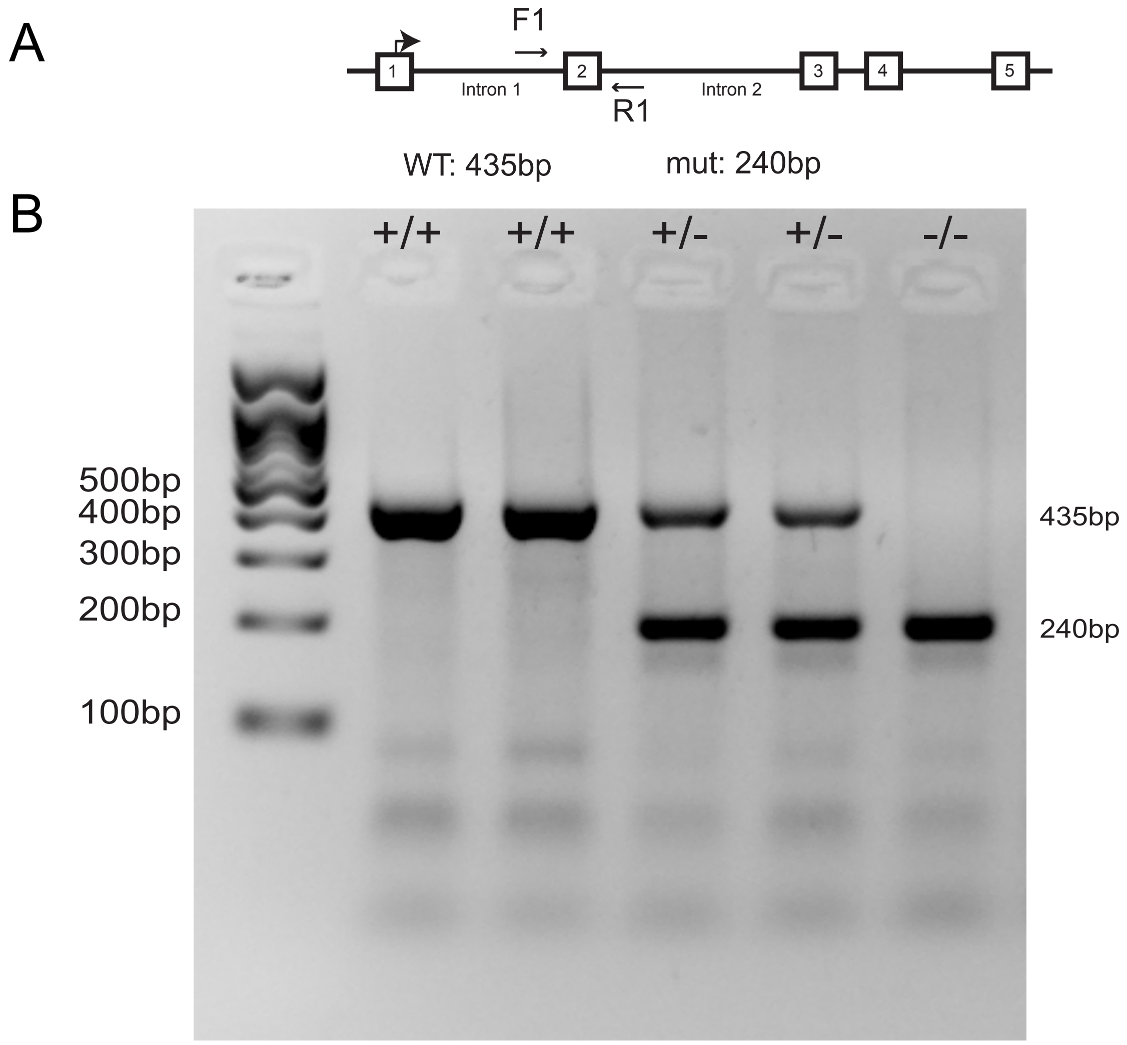

Supplementary Figure 12. Genotyping of the *Snap29* deletions.
A. Schematic representation of the *Snap29* locus with the primer (F1, R1) used for genotyping. B. Example of PCR genotyping. The WT 435bp and the mutant 240bp are detected in the heterozygotes.

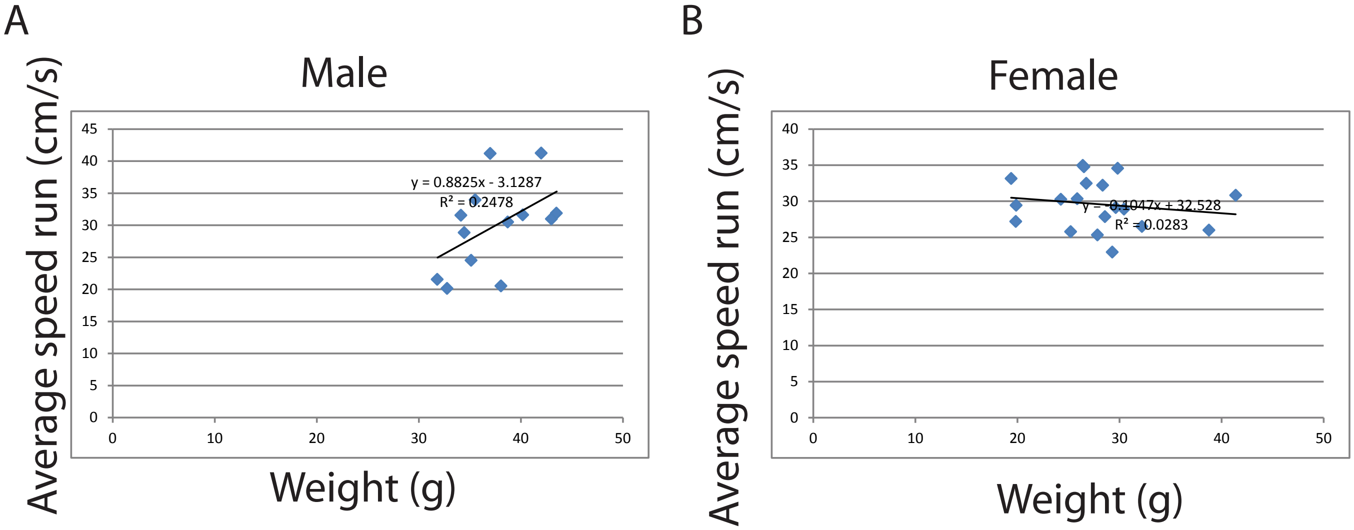

Supplementary Figure 13. Weight and average run speed are not correlated.
Plot showing that weight and the speed of male mice are not correlated (R2:0.2478) (A). Similarly, the weight and the speed of female mice are not correlated (B) (R2: 0.0283).

Supplementary Table 1

Snap29 skin abnormalities in P1-P7 pups

Genotype	none (died)	mild (died)	moderate (died)	severe (died)
-/-	7 (2)	14 (1)	14 (8)	5 (3)
+/-	0	0	0	0
+/+	0	0	0	0

Supplementary Table 2

Barrier defects in *Snap29* E12.5 embryos

barrier defects	+/+	+/-	-/-
none	7	9	3
partial	3	8	12
full	0	0	0

Supplementary Table 3

Catwalk analysis of *Snap29* mutant animals

	male					female					
	animal	RF	RH	LF	LH	animal	RF	RH	LF	LH	
Average speed	n.s.	n.a.	n.a.	n.a.	n.a.	0.0185↓	n.a.	n.a.	n.a.	n.a.	**run characterization**
Initial dual stance	n.a.	n.s.	n.s.	n.s.	n.s.	n.a.	0.0002↑	n.s.	0.0002↑	n.s.	**temporal**
Terminal dual stance	n.a.	n.s.	n.s.	n.s.	n.s.	n.a.	<0.0001 ↑	n.s.	<0.0001↑	n.s.	
Print length	n.a.	n.s.	n.s.	n.s.	n.s.	n.a.	0.0011↑	n.s.	0.0011↑	n.s.	**Spatial**
Swing speed	n.a.	n.s.	n.s.	0.0179↓	0.0179↓	n.a.	0.0009↓	0.000 9↓	0.0009↓	0.0009↓	**Kinetic**
Body speed	n.a.	0.0129↓	0.0129↓	0.0129↓	0.0129↓	n.a.	0.002↓	0.002 ↓	0.002↓	0.002↓	
Support two paws	0.019↓	n.a.	n.a.	n.a.	n.a.	0.0202↓	n.a.	n.a.	n.a.	n.a.	**Interlimb coordination**
Support three	0.0404↑	n.a.	n.a.	n.a.	n.a.	n.s.	n.a.	n.a.	n.a.	n.a.	
BOS_FrontPaws	n.s.	n.a.	n.a.	n.a.	n.a.	0.022*↑	n.a.	n.a.	n.a.	n.a.	
PhasedispersionLF_LH_Cstat	0.0172↑	n.a.	n.a.	n.a.	n.a.	n.s.	n.a.	n.a.	n.a.	n.a.	
Phasedispersion_LF_RH_CStat	n.s.	n.a.	n.a.	n.a.	n.a.	0.0297↑	n.a.	n.a.	n.a.	n.a.	
Couplings_LH_LF_Cstat	0.0177↓	n.a.	n.a.	n.a.	n.a.	n.s.	n.a.	n.a.	n.a.	n.a.	
Couplings_LF_LH_Cstat	0.0256↑	n.a.	n.a.	n.a.	n.a.	n.s.	n.a.	n.a.	n.a.	n.a.	

* difference is between +/+ vs +/-   n.s.= not significant   ↑=up   n.a.= not applicable   ↓=down

RH=Right hindlimb   LH= Left hindlimb   RF= Right forelimb   LF= Left forelimb

Supplementary Table 4

ERG b wave amplitude average

	+/+ (n=5)	+/- (n=8)	-/- (n=3 with abnormal ERG)	-/- (n=5)
Scotopic	628.97±113.45μV	654.31±42.22μV	**319.89±111.44μV**	672.23±154.04μV
Photopic	107.82±35.6μV	111.77±12.25μV	**20.95±32μV**	88.3±12.68μV

Supplementary Table 5

Scored parameters for the organs of male *Snap29* mice. Different letters denote significant differences for comparison of each organ among the three groups (Kruskal-Wallis ANOVA, p≤0.05, n=6-8)

parameters	+/+	+/-	-/-
Testis weight ($x10^{-2}$g/bw)	0.54±0.07[a]	0.50±0.12[a]	0.29±0.06[b]
Epididymis weight ($x10^{-2}$g/bw)	0.20±0.03[a]	0.23±0.02[a]	0.22±0.01[a]
Seminal vesicles ($x10^{-2}$g/bw)	0.79±0.06[a]	0.98±0.17[a]	0.72±0.08[a]
Prostate ($x10^{-2}$g/bw)	0.19±0.05[a]	0.19±0.00[a]	0.17±0.03[a]
Coagulating glands ($x10^{-2}$g/bw)	0.10±0.01[a]	0.13±0.04[a]	0.07±0.01[a]
Abnormal seminiferous tubules (%)	0.15±0.10[a]	2.23±1.06[a,b]	10.31±3.67[b]

Supplementary Notes

Snap29 homozygous mutant mice move with reduced speed and show forelimb preferences

We assessed locomotion and coordination of 6-weeks old male and female mice using the CatWalk system[1] and found significant differences in several parameters assessed on the Catwalk when and female *Snap29* homozygous mutants (n=14) were compared to female control littermates (n=12 wildtype and n=14 heterozygous). Specifically, average speed was significantly reduced in female *Snap29* homozygous mutants, when compared to female wild type litter mates (Figure 5A; p= 0.0185, ANOVA). In addition, initial dual stance - time of the initial step in each Step Cycle when both front or hind paws simultaneously make contact with the glass plate (Figure 5B; p= 0.0002) and terminal dual stance (the second step of each Step Cycle) (Supplementary Figure 7A; p= <0.0001) were significantly increased in forelimbs of female *Snap29* homozygous mutants. Also, print length - the distance between two prints was significantly increased in forelimbs of female homozygous mutants (Figure 5C; p = 0.0011). Furthermore, significant decreases were found in swing speed (Figure 5D; p=0.0009) and body speed (Supplementary Figure 7B; p =0.0129) of female *Snap29* homozygous mutants. Finally, diagonal support on two paws was significantly decreased (Figure 5E; p= 0.0202), whereas BOS front pwas and Phase Dispersion Left hindlimb (LF) and right hindlimb (RH) Cstat were significantly increased in female homozygous mutants (Supplementary Figure 6C-D; p=0.0297).

Similar changes were found when *Snap29* homozygous mutant males, (n=9) were compared to sex-matched controls (n= 11 wild type, n=9 for heterozygous). Specifically, velocity was significantly reduced in both forelimbs and in the left hindlimb (Figure 6A; p<0.05), while body speed was significantly decreased in all paws of homozygous mutant males (Supplementary Figure 8A; p =0.0129). Furthermore, diagonal support on two paws was significantly decreased (Figure 6B p=0.0202), while support on three paws was significantly increased in *Snap29* homozygous mutant males (Supplementary Figure 8B; p= 0.019). Also, *Snap29* homozygous mutant males displayed significant increases in Phase Dispersion LF_LH Cstat, (Supplementary Figure 8C; p=0.0172), and Cstat of couplings LF_LH, (Supplementary Figure 8D; p=0.0177), when compared with controls. Consistent with these observations Cstat for couplings LH_LF was significantly decreased in homozygous mutant males (Supplementary Figure 8E; p=0.0256), when compared to controls. Altogether, the Catwalk analysis indicate that

Snap29 homozygous mutant female and male mice move slower than their littermate controls and prefer to use their forelimbs for movement.

Snap29 homozygous mutant embryos show normal neurogenesis and lamination of the cerebrum

Since patients with CEDNIK show severe structural abnormalities in the brain, and given that *Snap29* mutant mice exhibited motor defects without skeletal malformations in their limbs (data not shown), we first assessed if these mice exhibited brain defects. We tested if neurons were either not produced or were not proliferating in *Snap29* embryonic brain. We evaluated proliferation of neurons by injecting pregnant females with BrdU at E13.5, followed by EdU at E15.5 and performing confocal microscopy to detect new incorporation of those DNA analogs *in vivo* at E17.5 (Supplementary Figure 9). We observed increased labelling of BrdU and EdU in one of four *Snap29* homozygous mutant embryos examined (Supplementary Figure 9B-B'''), when compared to wild-type siblings (Supplementary Figure 9A-A'''). Thus, we surmised that proliferation may be perturbed in a small subset of *Snap29* homozygous mutant mice but with low penetrance.

To determine if changes in proliferation result in perturbed neurogenesis, cortical lamination was examined. Cortical lamination was evaluated using molecular markers in wild type and *Snap29* homozygous mutant embryos at E17.5. The adult cerebral cortex consists of six primary layers, I-VI, from outside (pial surface) to inside (white matter); layer I is the upper most layer adjacent to the pial surface and layer VI is the deepest layer bordering the white matter (Supplementary Figure 10A). Molecular markers, known to label neurons in specific layers (reviewed in[2]), were used to evaluate the number of neurons in select cortical layers. For example, CTIP2 and TBR1 label neurons in deeper cortical layers (CTIP2 predominantly layer V, with some labeling in layer VI; TBR1for the subplate, layer VI, and some neurons in layer V), while SATB2 and REELIN label neurons in upper cortical layers (SATB2 is for layers II/III and IV/V; REELIN for layer I)[2,3]. Using antibodies against these four proteins, we compared the lamination of *Snap29* homozygous mutant cortex to that of wild type litter mates. No significant differences were found between the two genotypes (Supplementary Figure 10B-E). Thus, cerebral abnormalities found in CEDNIK are not modeled in *Snap29* homozygous mutant mice on a mixed genetic background.

However, as we serendipitously observed seizures in a subset of *Snap29* homozygous mutant mice at P10 (N=2/40; Supplementary Video 2), we postulated that these animals may have cerebral malformations but at very reduced penetrance or suffer from neuronal degeneration. Therefore, MRI was used to examine brains of 6-weeks old wild type (n=2) and *Snap29* homozygous mutants (n= 3) mice (Supplementary Figure 11). However, no significant differences were found, suggesting that severe brain malformations are not responsible for motor defects found in *Snap29* homozygous mutant mice.

Supplementary Methods

CatWalk Automated Quantitative Gait Analysis

CatWalk program (CatWalk XT 10.6, Noldus, Leesburg, VA. USA) was used to analyze the gait of the mice according to manufactures instructions and published procedures[4]. Animals were trained for 3 days before the final measurements were collected. A minimum of 3 compliant runs were acquired with following run Criteria: Min Duration: 0.5 sec, Max Duration: 8 secs, Max Variation: 60%, Min Number of Compliant Runs: 3. Parameters of acquisition are as following: Camera Gain: 15 db, Green Intensity Threshold: 0.3, Red Ceiling Light: 17.4 V, Green Walkway Light: 16 V, Camera Position: 24 cm from the glass. Mean of all compliant runs per mouse were used to calculate gate of mice within different groups, according to sex and genotype. Data acquired on the experimental day were compared for each genotype. All five categories of gait parameters classified in[1] (parameters related to individual paws, the position of footprints, and time dependent relationship between footprints) were assessed. Parameters showing differences on the experimental day were also compared for each training day (day1-3). To include data for different trial days, the average of total measurements (wildtype, heterozygous and mutant) for each parameter on a given day was acquired. Each measurement was then divided by the experimental average and the normalized data from different experiments were pooled together before statistical analysis. We found no correlation between the weight of mice and parameters related to speed (Supplementary Figure 13), therefore, experimental data were adjusted by normalizing against experimental average for each parameter or for each paw per parameters. Furthermore, as speed can be a compounding factor in these studies, we analyzed and confirmed that the average speed of all animals used for analysis was at

speeds that correlates to a "walk" gait as defined by Bellarfita and Kiehn (2015)[5]. All final results indicate parameters that showed a significant difference over all 4 days.

Cell counting

Two serial sections from each E17.5 wildtype (n=3) or *Snap29* homozygous mutant (n=4) embryo were used for quantification of each cortical layer marker (total 6 sections for wildtype and 8 sections for mutant). 10x images were taken from the cortical sections (at the level of internal capsule), and a selected area (200 μm wide) from each image as shown in Supplementary Figure 11A with a grid used for cell counting. As embryonic brains have less well-defined layer structures, instead of using layer numbers we used a grid system that divides the embryonic cortical neuroepithelium into eight parts (each with equal distance; grid 1 is closest to the pial surface and grid 8 is closest to the subventricular zone) for quantification (Supplementary Figure 11B-E). Each selected area is divided into 8 sub areas (divided with uniformly spaced lines horizontal to the ventricular surface) spanning from the pial surface to the intermediate zone (Grids 1-8; 1 being closest to the pial surface) excluding the ventricular zone and subventricular zone. TBR1, CTIP2, SATB2 positive cells were counted using Automatic Threshold (Yen) in Image J and Reelin positive cells were counted using Manual Threshold (Yen, set value: 215). Total cells (Hematoxylin positive) were counted using Automatic Local Threshold (Otsu, Radius:10). The percentage of TBR1+/total, CTIP2+/total, or SATB2+/total cells was plotted for each 8 Grid for wildtype or homozygous mutant. Error bars are Mean +/- SEM. The percentage of Reelin/total cells was plotted for Grid 1 (Error bars are Mean +/- SD). Multiple pairwise t tests were performed in Prism 8. The differences between wildtype and homozygous mutants are statistically not significant for all the layer markers tested here.

BrdU and EdU labeling and staining

Pregnant mice were injected with BrdU (50 mg/kg, i.p.) at E12.5 and E13.5, and with Edu injection (50 mg/kg, i.p.) at E14.5 and E15.5, respectively. Embryos were collected at E17.5 and fixed in 4%PFA for 48 hours. Paraffin sections (E17.5, coronal, 6 μm) were used for BrdU and EdU double staining. After deparaffinization and rehydration, antigen retrieval was performed as described above. The sections were first treated with 2N HCl for 30min at RT, then with 0.1M sodium tetraborate for 10 min, and finally with 0.5% Triton X-100 for 10 min. After

blocking with 10% goat serum for 1 hr, primary antibody, anti-BrdU antibody (Abcam, ab6326, 1:100 in 1% goat serum/PBS), was incubated for 4°C overnight on each section. Goat anti-rat IgG-Alexa Fluor 594 (1:200, Invitrogen) was applied as a secondary antibody for 1hr, followed by Click-iT reaction cocktail (Alexa Fluor 488, Invitrogen, C10337) for 30 min according to manufacturer's recommendation. Hoechst 33342 was used for nuclear staining.

Supplementary References

1 Hamers, F. P., Koopmans, G. C. & Joosten, E. A. CatWalk-assisted gait analysis in the assessment of spinal cord injury. *J Neurotrauma* **23**, 537-548, doi:10.1089/neu.2006.23.537 (2006).

2 Molyneaux, B. J., Arlotta, P., Menezes, J. R. & Macklis, J. D. Neuronal subtype specification in the cerebral cortex. *Nat Rev Neurosci* **8**, 427-437, doi:10.1038/nrn2151 (2007).

3 Colasante, G. *et al.* ARX regulates cortical intermediate progenitor cell expansion and upper layer neuron formation through repression of Cdkn1c. *Cereb Cortex* **25**, 322-335, doi:10.1093/cercor/bht222 (2015).

4 Hamers, F. P., Lankhorst, A. J., van Laar, T. J., Veldhuis, W. B. & Gispen, W. H. Automated quantitative gait analysis during overground locomotion in the rat: its application to spinal cord contusion and transection injuries. *J Neurotrauma* **18**, 187-201, doi:10.1089/08977150150502613 (2001).

5 Bellardita, C. & Kiehn, O. Phenotypic characterization of speed-associated gait changes in mice reveals modular organization of locomotor networks. *Curr Biol* **25**, 1426-1436, doi:10.1016/j.cub.2015.04.005 (2015).
