# Supplementary material for: Snap29 mutant mice recapitulate neurological and ophthalmological abnormalities associated with 22q11 and CEDNIK syndrome
Source: Commun Biol. 2019 Oct 11;2:375. doi: 10.1038/s42003-019-0601-5 (PMC6789041; doi:10.1038/s42003-019-0601-5)
Supplement: Supplementary file 2 — Description of Additional Supplementary Files [file 42003_2019_601_MOESM2_ESM.docx]

**Supplementary** **Movie 1. *Snap29^-/-^* pups have difficulty turning back on their paws.** One homozygous mutant and one wild type *Snap29* pup is shown. Although both start on their back, the *Snap29^-/-^* pup is having difficulty turning back on its paws. Also, once it righted itself, it immediately fell on its back.

**Supplementary Movie 2. *Snap29^-/-^* mice exhibiting seizures.** Two of the three mice exhibited seizures as measured by episode of intense shaking. These animals turned out to be *Snap29^-/-^* after genotyping.

**Supplementary Data 1:** Excel file containing the data used to generate the graphs presented in the mains Figure 1D, 5 and 6.
