# Supplementary material for: Snap29 mutant mice recapitulate neurological and ophthalmological abnormalities associated with 22q11 and CEDNIK syndrome
Source: Commun Biol. 2019 Oct 11;2:375. doi: 10.1038/s42003-019-0601-5 (PMC6789041; doi:10.1038/s42003-019-0601-5)
Supplement: Supplementary file 6 — Reporting Summary [file 42003_2019_601_MOESM6_ESM.pdf]

nature research

Corresponding author(s): Loydie Jerome-Majewska

Last updated by author(s): Aug 20, 2019

Reporting Summary

Nature Research wishes to improve the reproducibility of the work that we publish. This form provides structure for consistency and transparency in reporting. For further information on Nature Research policies, see Authors & Referees and the Editorial Policy Checklist.

Statistics

For all statistical analyses, confirm that the following items are present in the figure legend, table legend, main text, or Methods section.

n/a	Confirmed	
☐	☒	The exact sample size (*n*) for each experimental group/condition, given as a discrete number and unit of measurement
☐	☒	A statement on whether measurements were taken from distinct samples or whether the same sample was measured repeatedly
☐	☒	The statistical test(s) used AND whether they are one- or two-sided<br>*Only common tests should be described solely by name; describe more complex techniques in the Methods section.*
☐	☒	A description of all covariates tested
☐	☒	A description of any assumptions or corrections, such as tests of normality and adjustment for multiple comparisons
☐	☒	A full description of the statistical parameters including central tendency (e.g. means) or other basic estimates (e.g. regression coefficient) AND variation (e.g. standard deviation) or associated estimates of uncertainty (e.g. confidence intervals)
☐	☒	For null hypothesis testing, the test statistic (e.g. *F*, *t*, *r*) with confidence intervals, effect sizes, degrees of freedom and *P* value noted<br>*Give P values as exact values whenever suitable.*
☒	☐	For Bayesian analysis, information on the choice of priors and Markov chain Monte Carlo settings
☒	☐	For hierarchical and complex designs, identification of the appropriate level for tests and full reporting of outcomes
☐	☒	Estimates of effect sizes (e.g. Cohen's *d*, Pearson's *r*), indicating how they were calculated

Our web collection on statistics for biologists contains articles on many of the points above.

Software and code

Policy information about availability of computer code

Data collection	Excel, Adobe Photoshop and Illustrator, Image J.
Data analysis	Prism 5 GraphPadVersion 1.0.0.0 was used to analyzed. No custom codes were genrated.

For manuscripts utilizing custom algorithms or software that are central to the research but not yet described in published literature, software must be made available to editors/reviewers. We strongly encourage code deposition in a community repository (e.g. GitHub). See the Nature Research guidelines for submitting code & software for further information.

Data

Policy information about availability of data

All manuscripts must include a data availability statement. This statement should provide the following information, where applicable:
- Accession codes, unique identifiers, or web links for publicly available datasets
- A list of figures that have associated raw data
- A description of any restrictions on data availability

All relevant data generated or analysed during this study are included in this article. Supplementary Data is available upon reasonable request from the corresponding author.

Field-specific reporting

Please select the one below that is the best fit for your research. If you are not sure, read the appropriate sections before making your selection.

☒ Life sciences ☐ Behavioural & social sciences ☐ Ecological, evolutionary & environmental sciences

Life sciences study design

All studies must disclose on these points even when the disclosure is negative.

Sample size	A minimum of three animals per sex and per genotype were analyzed per experiments. These animals came from at least 3 different litters to test for reproducibility.
Data exclusions	No data were excluded from analysis
Replication	All data were replicatable from litter to litter.
Randomization	Samples were distributed into wild-type, heterozygous or homozygous mutant groups based on PCR-genotype, as explained in the methods section. Male and female groups were distributed based visual sexing of mice, or PCR sexing of fetus.
Blinding	Experimenters were not aware of the genotyping while performing experiments.

Reporting for specific materials, systems and methods

We require information from authors about some types of materials, experimental systems and methods used in many studies. Here, indicate whether each material, system or method listed is relevant to your study. If you are not sure if a list item applies to your research, read the appropriate section before selecting a response.

Materials & experimental systems

n/a	Involved in the study	
☐	☒	Antibodies
☒	☐	Eukaryotic cell lines
☒	☐	Palaeontology
☐	☒	Animals and other organisms
☒	☐	Human research participants
☒	☐	Clinical data

Methods

n/a	Involved in the study	
☒	☐	ChIP-seq
☒	☐	Flow cytometry
☒	☐	MRI-based neuroimaging

Antibodies

Antibodies used

1. SNAP29 monoclonal antibody (1:5000, Abcam, ab138500) clone EPR9199.
2. rabbit anti-Beta-actin monoclonal antibody (1:5000, Cell Signaling, #4970) clone 13E5
3. anti-rabbit secondary antibody (1:5000, Cell Signaling, 7074)
4. TBR1 (rabbit, 1:1000, Abcam, ab31940)
5. CTIP2 (rat, 1:200, Abcam, ab18465) clone 25B6.
6. Reelin (mouse, 1:200, Millipore, MAB5364) clone G10
7. SATB2 (mouse, 1:200, Bio Matrix Research, BMR00263)

Validation

1. Validated for WB and IP. Deletion of exon 2 leads to a loss of signal in homozygous mutant tissues and a reduction of signals in the heterozygotes (Figure 1).
2. Validated using flow cytometry (Flow Cyt), immunohistochemistry (IHC), immunofluorescence (IF) and western blot (WB).. Does not recognized skeletal, cardiac, vascular smooth and enteric smooth muscle isoforms.
4.Validated for Flow Cyt, IHC, IF, WB.
5. Validated for Flow Cyt, IHC, IF, WB.
6. Validated for IHC and WB
7. Validated for WB and IP and IF, IHC.

Animals and other organisms

Policy information about studies involving animals; ARRIVE guidelines recommended for reporting animal research

Laboratory animals	CD1 mice were purchased from Charles River laboratories. Snap29 mutant animals were generated in-house
Wild animals	Not applicable
Field-collected samples	Not applicable
Ethics oversight	All procedures and experiments were performed according to the guidelines of the Canadian Council on Animal Care and

Ethics oversight	approved by the Animal Care Committee of the RI-MUHC.

Note that full information on the approval of the study protocol must also be provided in the manuscript.
